# Surgical treatment of congenital biliary duct cyst

**DOI:** 10.1186/1471-230X-12-29

**Published:** 2012-03-30

**Authors:** De-chun Wang, Zi-pei Liu, Zhi-hua Li, Da-jiang Li, Jian Chen, Shu-guo Zheng, Yu He, Ping Bie, Shu-guang Wang

**Affiliations:** 1 Institute of Hepatobiliary Surgery of PLA, Southwest Hospital, Third Military Medical University, Chongqing 400038, China

**Keywords:** Congenital biliary duct cyst, Total cyst excision, Laparoscopic resection

## Abstract

**Background:**

It is acknowledged that total cyst excision is a safe and ideal surgical treatment for congenital biliary duct cyst, compared to simple internal drainage. The aim of this study was to determine the optimal operation occasion and the effect of laparoscopy on congenital biliary duct cyst based upon total cyst excision.

**Methods:**

From January 2002 to January 2011, 217 patients were admitted to Southwest Hospital for congenital biliary duct cyst. To determine the optimal surgery occasion, we divided these subjects into three groups, the infant group (age ≤ 3 years), the immaturity group (3 < age ≤ 18 years), and the maturity group (age > 18 years), and then evaluated the feasibility, risk and long-term outcome after surgery in the three groups. To analyze the effect of laparoscopic technique on congenital biliary duct cyst, we divided the patients into the laparoscopy and the open surgery groups.

**Results:**

Among the three groups, the morbidity from cholangiolithiasis before surgical treatment had obvious discrepancy (p < 0.05) (lowest in the infant group), and intraoperative blood loss also had apparent diversity (p < 0.05). Furthermore, long-term outcomes (secondary cholangiolithiasis, stoma stenosis and cholangiocarcinoma) showed no significant difference between different groups (p > 0.05).

Similarly, no significant discrepancy was observed in the morbidity from postoperative complications or long-term postoperative complications (p > 0.05) between the laparoscopic and the open surgery groups.

**Conclusions:**

We conclude that total cyst excision should be performed as early as possible. The optimal treatment occasion is the infant period, and laparoscopic resection may be a new safe and feasible minimally invasive surgery for this disease.

## Background

**Figure 1:**
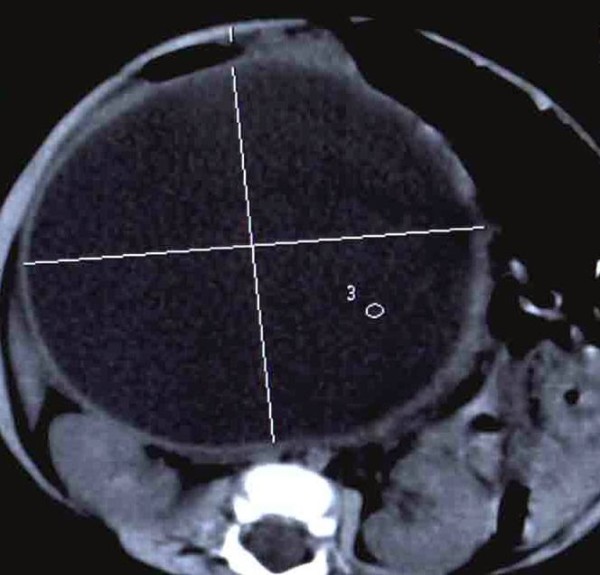
**This CT scan shows irregular dilatation of common bile**.

Congenital biliary duct cysts are congenital anomalies of the biliary tree which are characterized by cystic dilatation of the extra- and/or intra-hepatic biliary ducts. The classic clinical symptoms of the cyst are jaundice, right upper quadrant pain, and palpable abdominal masses. Unlike Western countries, the morbidity of congenital bile duct cyst is much higher in Asia [1,2]. Years ago, the primary treatment of cysts was simple internal drainage by cyst-enterostomy or partial cyst excision. However, several serious clinical outcomes, including stomal stenosis, cholestasis, cholangiolithiasis, and even cholangiocarcinoma, led to poor prognosis, and even secondary surgical operation [3]. At present, total cyst excision with Roux-en-Y cholangiojejunostomy is considered to be a safer and more ideal treatment for patients with congenital biliary duct cysts [4]. The surgical approaches depend on cyst clinical typing [5,6], which has been properly demonstrated by imaging findings (Figure 1), such as computed tomography (CT), magnetic resonance cholangiopancreatography (MRCP), endoscopic retrograde cholangiography (ERCP), etc [7-9]. Appropriate management of types I and II choledochal cysts include cholecystectomy, entire resection of the extrahepatic biliary tract with choledochal cyst, and Roux-en-Y cholangiojejunostomy. Resection of the extrahepatic biliary tract is also recommended for type IV cyst. As for type IV cyst, if there are stricture and/or stones in the intrahepatic abnormal biliary duct, and the cysts are confined to one lobe (or segment), hepatic lobectomy may also be considered. Bipolar intrahepatic cysts are associated with a high risk of intrahepatic stones and are managed with long-term transhepatic stenting to provide continuous access to the intrahepatic biliary tree for stone retrieval [2]. The treatment of type V cyst also depends on its localization [10]. Congenital bile duct cysts are commonly diagnosed in young women (the female to male ratio is 3: 1) [11]. As these patients value the cosmetic results as well as cure of the disease, minimally invasive surgery becomes more prevalent and appropriate. In recent years, laparoscopic resection has been applied for congenital bile duct cysts [12]. The congenital bile duct cysts have a long course of disease, which may lead to increasing morbidity of several complications, including cholestatis, cholangiolithiasis, and even cholangiocarcinoma. However, operation at childhood may increase life-threatening risks. Thus, the best surgical timing for this disease remains controversial.

In the present study, we will investigate the optimal timing of total cyst resection and the effect of laparoscopic technique on the disease.

## Methods

### Patients

From January 2002 to January 2011, 217 patients were admitted to Southwest Hospital, (Chongqing, China) for congenital biliary duct cyst. Demographic data and preoperative status were collected prospectively and analyzed retrospectively. Of the 217 patients, 187 consecutive cases underwent total cyst excision in our hospital. Data were collected retrospectively from a computerized database and analysis of patients' hospital charts. Supplementary information was obtained from standardized telephone interviews with the patients during the follow-up. This study was approved by the Ethical Committee of Southwest Hospital.

### Preoperative evaluation

Parameters of the hepatobiliary system were evaluated preoperatively, including demography, age, gender, clinical symptoms, biliary complication, localization of cyst, and imaging findings (ultrasonography, computed tomography (CT) scan, magnetic resonance cholangiopancreatography (MRCP), and endoscopic retrograde cholangiopancreatography (ERCP)).

### Postoperative management

All the patients received the same postoperative management by the same team of surgeons. Patients who received surgical treatment were monitored in the intensive care unit (ICU) during the early postoperative period. Subsequently, whether to stay in the ICU depended on the patient's condition.

### Surgical treatment

No type III cyst patient was included in the 187 patients who underwent total cyst excision in our hospital. Treatment of cyst depended on clinical typing. Patients with types I and II cyst underwent total extra-hepatic cyst excision. The division of the biliary tract should be connected to healthy biliary tissues either in the superior or the inferior part of the cyst. Meanwhile, the Roux-en-Y cholangiojejunostomy was also conducted. In this study, the intra-hepatic cysts of types IV and V were localized forms. Thus, patients with type IV cyst also needed total extra-hepatic cyst excision and Roux-en-Y cholangiojejunostomy. If intra-hepatic cysts were localized and complicated with biliary ductal stricture, stones, abscess, or liver atrophy, the partial hepatectomy would be carried out for type IV cysts. Treatment of patients with type V cyst also relied on the location of bile duct abnormalities. Patients with type V cyst should undergo partial hepatectomy and Roux-en-Y cholangiojejunostomy, if complicated with biliary ductal stricture, stones, abscess, or liver atrophy. Otherwise, they only received Roux-en-Y cholangiojejunostomy.

### Follow-up

All the patients were followed up by the same team of surgeons after the surgery every 2 to 3 months. Ultrasonography, ERCP, or MRCP was annually conducted for stenosis, stone, or even advanced tumor. Patients with stenosis or cholangiolithiasis were treated conservatively if they had no or mild symptoms. Patients with cholangiocarcinoma were suggested with radical resection. If the patients with cholangiolithiasis and severe life-threatening attack of acute cholangitis did not respond to initial conservative treatments, other definitive operations should be considered.

### Statistical analysis

Statistical analysis was performed using SPSS 18. If there were 2 or more expectations less than 5, continuous data would be assayed with Fisher exact test; otherwise, data were analyzed with chi-aquare and t tests. 

## Results

**Figure 2:**
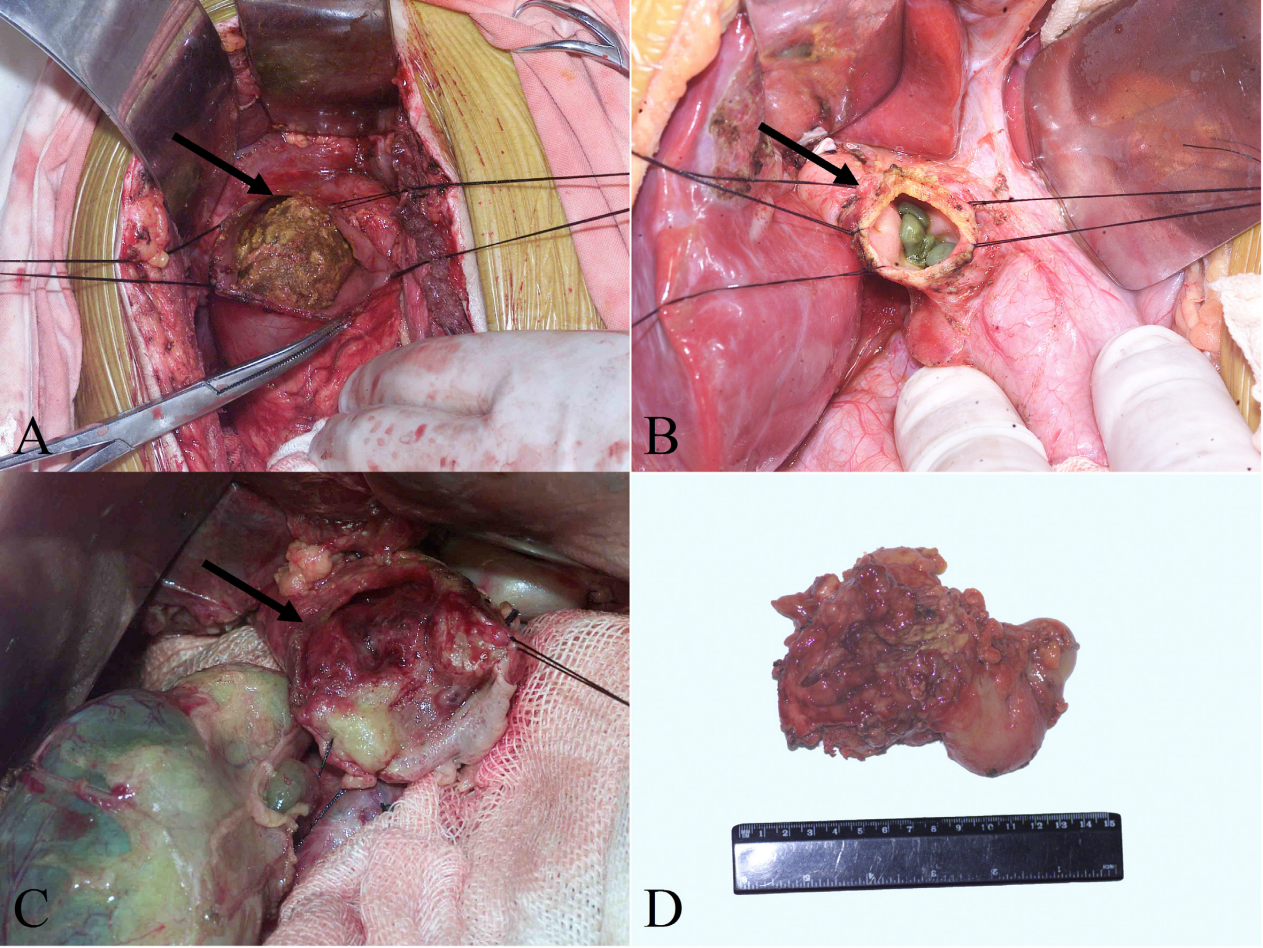
**The gross images show the complications of the bile duct cyst specimen**. (A-B) These images show stones in the cyst (arrows), (C-D) These images show the cancer of cysts (arrow).

The demographic data and preoperative status of the 217 patients are shown in Table 1. Of all the patients, the ratio of female to male was 170: 47 (3.6: 1), and the age ranged from 4 months to 80 years with a mean age of 28.20 years. And the most common symptom was **stomachache**. Among the patients, 80 had intra- and/or extra-cholangiolithiasis before treatment (Figure 2A-B), and 8 had advanced cholangiocarcinoma (Figure 2C-D).

**Table 1:** Demographic Data and Preoperative Status

Characteristics	Data
*Age (years)*	28.2 ± 18.8

*Gender*	

Female, n (%)	170 (78.3%)

Male, n (%)	47 (21.7%)

*Presenting symptoms*	212 (97.7%)

Abdominal pain, n (%)	191 (90.1%)

Jaundice, n (%)	57 (26.9%)

Cholangitis, n (%)	23 (10.8%)

*Clinical typing*	

Type I, n (%)	161 (74.2%)

Type II, n (%)	1 (0.4%)

Type IV, n (%)	36 (16.6%)

Type V, n (%)	19 (8.8%)

### Total cyst excision should be performed as early as possible, and the optimal treatment time is the infant period

Of 80 patients who had intra- and/or extra-cholangiolithiasis before surgical treatment, 2 belonged to the infant group (n = 23), 21 to the immaturity group (n = 58), and 57 to the maturity group (n = 136). The three groups showed significant discrepancy (p = 0.009 < 0.05) in cholangiolithiasis morbidity before surgical treatment. The cholangiolithiasis morbidities in the immaturity and maturity groups were significantly higher than that in the infant group (p = 0.005 < 0.05); however, no discrepancy existed between the immaturity and maturity groups (p > 0.05). In addition, 8 patients had cholangiocarcinoma, of whom 1 belonged to the immaturity group and 7 to the maturity group. However, no significant discrepancy was observed between the three groups (p = 0.459 > 0.05). 

A total of 187 consecutive patients underwent total cyst excision for congenital biliary duct cyst in our hospital (Table 2). The youngest patient aged 4 months and the oldest 75 years. Among the three groups, intraoperative blood loss was significantly different (p < 0.05), with the smallest value in the infant group (Table 3).

**Table 2:** Basic characteristics of patients underwent total cyst excision

Characteristics	Data
*Age (years)*	27.8 ± 18.2

*Gender*	

Female, n (%)	153 (81.8%)

Male, n (%)	34 (18.2%)

*Clinical typing*	

Type I, n (%)	147 (78.6%)

Type II, n (%)	1 (0.5%)

Type IV, n (%)	28 (15.0%)

Type V, n (%)	11 (5.9%)

**Table 3:** Intraoperative and postoperative characteristics of patients underwent total cyst excision

Group	Infant	Immaturity	Maturity
***Intraoperative***			

Blood loss (ml)	66.0 ± 34.2	261.4 ± 305.6*	389.4 ± 402.6*^#^

Operation time (min)	289.0 ± 70.4	341.2 ± 104.3	320.0 ± 120.4

***Postoperative***			

Hemorrhage (n)	0	0	4

Pulmonary infection (n)	3	0	4

Others (n)	2	1	7

* p = 0.006 < 0.05, compared with the infant in intraoperative blood loss

*p = 0.000 < 0.05, compared with the infant in intraoperative blood loss

# p = 0.046 < 0.05, compared with the immaturity in intraoperative blood loss

After the surgery, 153 of 187 patients having undergone total cyst excision were regularly followed up. The visited rate was 81.8%, and the visited duration was 10 to 116 months. There were 10 patients with secondary cholangiolithiasis, 4 with stoma stenosis, and 2 with secondary cholangiocarcinoma. Of them, none of the infant patients (n = 18) suffered from intra- and/or extra-cholangiolithiasis, stoma stenosis, or secondary cholangiocarcinoma. In contrast, 1 patient in the immaturity group (n = 46) had cholangiolithiasis, and in the maturity group (n = 89), 9 suffered from secondary cholangiolithiasis, 4 from stoma stenosis, and 2 from secondary cholangiocarcinoma. However, no significant discrepancy existed among the three groups in morbidity from cholangiolithiasis, stenosis or advanced tumors (p > 0.05).

### Totally laparoscopic resection may be a new safe and feasible minimally invasive surgery for this disease

Three patients in the laparoscopic group (n = 22) and 14 patients in the open surgery group (n = 165) showed postoperative complications. After a long-term follow-up, 13 patients in the open surgery group (n = 136) reported secondary cholangiolithiasis, stoma stenosis or cholangiocarcinoma, whereas no patient in laparoscopic group (n = 17) reported those diseases. However, no significant discrepancy was observed between the two groups in postoperative complications or long-term follow-up results (p = 0.668 > 0.05, p = 1.0 > 0.05, respectively).

## Discussion

Internal drainage by cyst-enterostomy or partial cyst excision was once considered the preferred surgical treatment for congenital bile duct cysts. However, its poor prognosis (frequent cholangitis, cholangiolithiasis, and even cholangiocarcinoma) confined its application severely [3]. In recent years, total cyst excision is regarded a more ideal and reasonable treatment for this disease. Our previous study has also confirmed that the total cyst excision has better prognosis than single internal drainage [13]. However, the optimal surgical treatment timing is still controversial.

Congenital bile duct cysts cause abnormal bile duct structure, which is the main factor for cholangiolithiasis and cholangiocarcinoma. The dilated bile ducts cause dilatation-stenosis-like biliary stricture, which subsequently leads to the abnormality of bile dynamics, causing cholestasis and then inducing biliary tract inflammation. In addition, cholestasis could further cause cholangitis and cholangiolithiasis [14], and even cholangiocarcinoma after long-term inflammatory stimulation [15]. The above evidence suggests that the longer the disease course is, the higher the morbidity of complications will be. This study also suggests that obvious discrepancy exists in cholangiolithiasis morbidity among three groups before the surgical treatment. Of all patients, eight suffer from advanced cholangiocarcinoma (the maturity = 7 and immaturity = 1), which did not arise in infancy. No significant difference was abserved among the three age groups, which may be explained by the limited period of observation.

**Figure 3:**
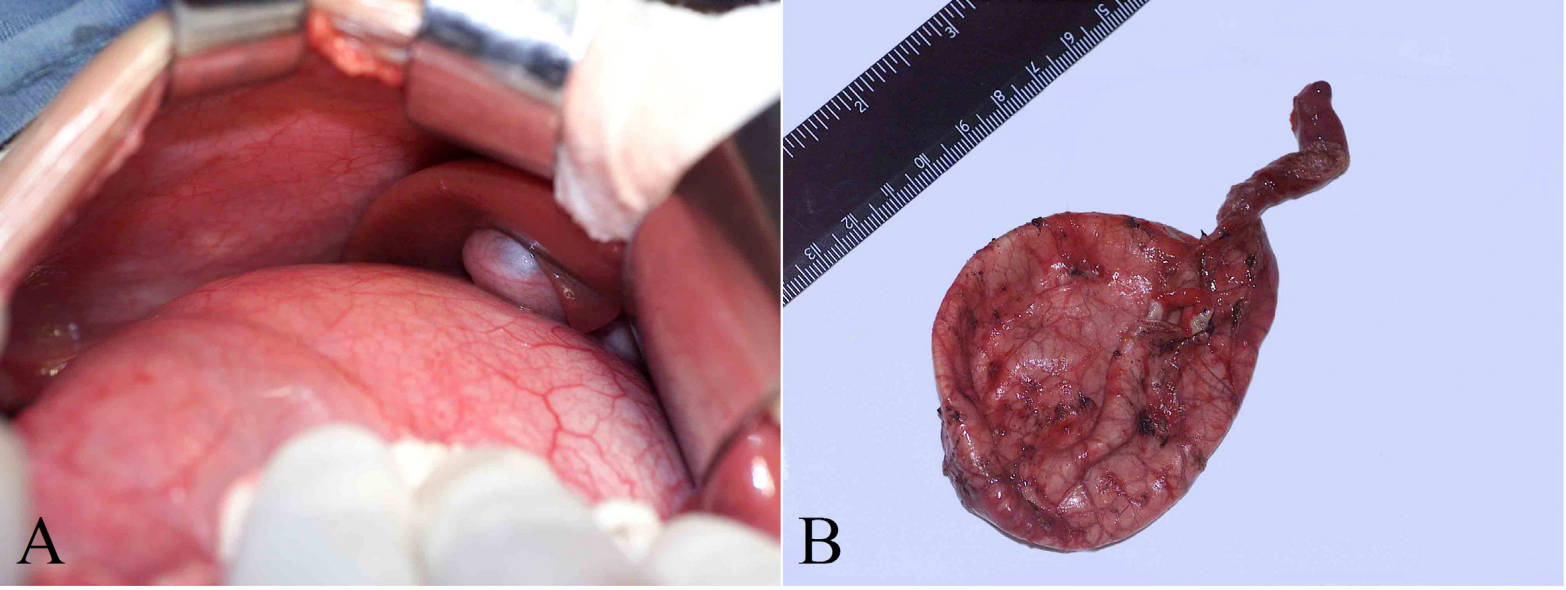
**These gross images of the bile duct cyst specimen**. (A) The image of the bile duct cyst in operation. (B) The specimen of the bile duct cyst.

At present, considering the growth and development of the patients and the surgical risks, most surgeons suggest that the infants should not undergo the surgical treatment until they have grown up. However, compared with the adults, the infants have advantages in receiving the surgery. For example, the infants have shorter disease course, which means much milder inflammation and less tissue adherence (Figure 3), which subsequently reduce the probability of hemorrhage and tissue injury, thus lowering the risk of operation. In this study, we have confirmed that the intraoperative blood loss is significantly different (p < 0.05) among the three groups, and the infant group has the lowest loss. Similarly, our long-term follow-up data have shown that the postoperative complications are not significantly different among the three groups. The above evidence suggests that the optimal treatment timing for congenital biliary duct cyst is in infancy, which causes the lowest risk. However, our data have shown a significant difference in the postoperative complications among the three groups (p = 0.012 < 0.05), with the morbidity of postoperative complications in the immaturity and maturity groups significantly lower than in the infant group (p = 0.036 < 0.05). Three patients in the infant group have complications. However, these postoperative complications are not lifethreatening and unresolvable, and they may be due to the difficulty in the respiratory management of infants, indicating that preventing pulmonary infection is very important after total cyst excision in infants.

**Figure 4:**
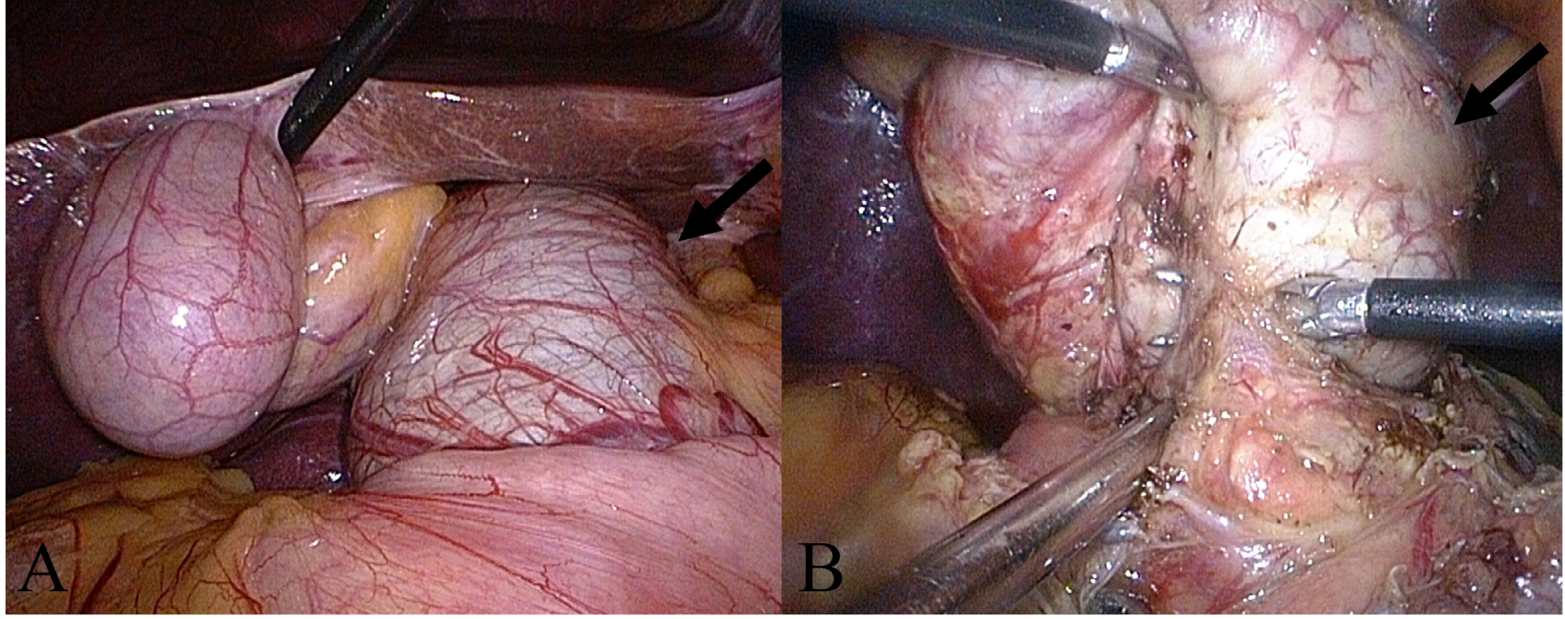
**These laparoscopic images show the bile duct cyst specimen**. (A) The image of cyst before operation (arrow), (B) The image of laparoscopic resection (arrow indicates cyst).

Patients with congenital biliary duct cysts are commonly young women, who value cosmetic results as well as cure of the disease. Thus, minimally invasive surgery is important. To date, totally laparoscopic resection, which is safer and more feasible, has been applied for congenital biliary duct cysts [12] (Figure 4). In addition, the laparoscopic technology can relieve and even eliminate tissue adhesion, and early postoperative pain, and can promote resumption of peristalsis, excellent esthetics, and resumption of activities. Furthermore, our data suggest that no significant difference exists in postoperative complications and long-term outcomes between the laparoscopic and open surgery groups. These findings suggest that totally laparoscopic resection for congenital bile duct cysts is feasible and causes low risk.

## Conclusions

In this study, we domonstrate that the infants with congenital bile duct cyst have lower morbidity of cholangiolithiasis before surgical treatment and lower intraoperative blood loss when undergoing total cyst excision than the immaturity and the maturity groups. In addition, long-term follow-up data indicate that there is no significant difference among the three groups. These findings suggest that total cyst excision should be performed as early as possible, the optimal treatment timing is during infancy, and the totally laparoscopic resection, which is safer and more feasible, may be a new minimally invasive surgery for this disease.

## Competing interests

The authors declare that they have no competing interests.

## Authors' contributions

DW participated in the design of the study, the analysis and interpretation of the data, and drafted the manuscript. ZL participated in the analysis and interpretation of the data. ZL, DL, JC, SZ, YH, PB, and SW performed the surgical procedures, and participated in the analysis and interpretation of the data. In addition, SW also performed the final approval of the article. All authors read and approved the final manuscript.

## Pre-publication history

The pre-publication history for this paper can be accessed here:

http://www.biomedcentral.com/1471-230X/12/29/prepub
